# Tackling the triple threats of childhood malnutrition

**DOI:** 10.1186/s12916-019-1464-9

**Published:** 2019-11-25

**Authors:** Martha Mwangome, Andrew M. Prentice

**Affiliations:** 1 KEMRI-Wellcome Trust Research Programme, Kilifi, Kenya; 2 MRC Unit The Gambia at London School of Hygiene & Tropical Medicine, Atlantic Boulevard, Fajara, Banjul, The Gambia

**Keywords:** Obesity, Stunting, Wasting, Nutrition, Risk factors

## Abstract

The term ‘double burden of malnutrition’ is usually interpreted in terms of the physical status of children: stunted and wasted children on the one hand and overweight/obese children on the other. There is a third category of malnutrition that can occur at either end of the anthropometric spectrum or, indeed, in children whose physical size may be close to ideal. This third type is most commonly articulated with the phrase ‘hidden hunger’ and is often illustrated by micronutrient deficiencies; thus, we refer to it here as ‘undernutrition’. As understanding of such issues advances, we realise that there is a myriad of factors that may be influencing a child’s road to nutritional health. In this *BMC Medicine* article collection we consider these influences and the impact they have, such as: the state of the child’s environment; the effect this has on their risk of, and responses to, infection and on their gut; the consequences of poor nutrition on cognition and brain development; the key drivers of the obesity epidemic across the globe; and how undernourishment can affect a child’s body composition. This collection showcases recent advances in the field, but likewise highlights ongoing challenges in the battle to achieve adequate nutrition for children across the globe.

## Background

The World Health Organization (WHO) has set a target to significantly reduce the childhood undernutrition and obesity ‘double burden of malnutrition’ by the year 2030 through working with lower- and middle-income countries (LMIC) to achieve food security (healthy foods) and boost disposable incomes [1]. In the era of the Millennium Development Goals, many countries met their targets of halving rates of childhood undernutrition but progress was patchy. Reductions in most Asian countries were spectacular, but progress in Africa was less encouraging and owing to population growth there was actually an increase in the total number of stunted children. At a global level this progress stalled in about 2014 and, as highlighted in the recent report *The State of Food Security and Nutrition in the World 2019* [2], is now deteriorating. In 2017, across the globe approximately 51 million children aged younger than 5 years were wasted (too light for their height) and 151 million were stunted, with the major proximal cause being poor nutrition. Conversely, 38 million were reported to be obese [3]. This special collection pulls together publications highlighting research into the mediating factors for, and potential solutions to, childhood undernutrition and obesity. They address a challenge that has faced public health and clinical nutritionists for decades: Can the rigorous application of research into causative pathways trigger a paradigm shift that will transform our currently limited ability to stimulate better growth and development in stunted and wasted children and ameliorate the dangers of excess adiposity at the other end of the spectrum? And can nations combat undernutrition without the anthropometric pendulum swinging too far and causing a new health burden in the form of childhood obesity?

## Challenges and possible solutions—a panacea remains elusive

Childhood stunting has been promoted as the most useful aggregate measure of chronic nutritional deficits in LMICs and has been adopted as a key metric for assessing progress towards the elimination of hunger [4]. Stunting is not just about an inadequate or nutrient-poor food supply; there are other contributing factors related to living in unhygienic environments. Persistent gut damage, termed ‘environmental enteric dysfunction’ (EED) [5], is considered a chief culprit and has been the target for numerous programmes aimed at reducing childhood stunting. We start the discussion of this collection with a review by Tickell et al. [6] summarising an important series of studies evaluating the causes and consequences of EED and outlining trials that have tested novel interventions to combat the condition. They present the latest insights into several of the long-recognised mechanisms and pathways. Intestines rendered permeable by EED allow bacteria to translocate into the systemic circulation, leading to subsequent immune activation and inflammation. Damaged intestinal structure (a commonly observed syndrome combining villous atrophy, crypt hyperplasia and a chronic inflammatory infiltrate) leads to a loss of absorptive surface area, nutrient leakage and energy wastage. EED may also cause enteric microbiome dysbiosis, leading to inflammation. The persistent challenge of finding reliable noninvasive measures of EED is raised and ongoing attempts to validate such assays against selected biopsies are listed [6]. This paper also tabulates 16 recently completed or ongoing trials aimed at treating EED. These cover anti-inflammatory drugs, antimicrobial interventions and dietary supplements.

Closely linked to EED is diarrhoeal disease, which has long been linked to all forms of childhood undernutrition. A study by Brander et al. [7] included in this collection provides further insights into the determinants of faltering linear growth among children with moderate-to-severe diarrhoea through a new analysis of data from the Global Enteric Multicenter Study. Aggregating data from the seven participating countries, the authors analysed predictors of linear growth failure in the 90 days following an episode of moderate-to-severe diarrhoea. A prediction model including (young) age, current stunting and wasting, presentation with a fever, or an Integrated Management of Childhood Illness danger sign yielded a receiver operating characteristic area under the curve value of 0.67, which unfortunately falls short of the thresholds that would normally be expected for a clinically useful prediction tool. This, together, with some of the putative predictors that failed to make it into the final model (e.g. unimproved sanitation and lower wealth) emphasises the wide spectrum of factors that contribute to EED and acute growth faltering and how difficult it is to single out individual remedial factors.

In the past 2 years, results have been published from three large trials aimed at ameliorating EED and stunting through the implementation of combined nutrition and water, sanitation and hygiene (WASH) interventions in Kenya and Bangladesh (the WASH Benefits trials [8, 9]) and in Zimbabwe (the SHINE trial [10]). These were supported by major investments from the Bill and Melinda Gates Foundation. Conducted to the highest standards, and with more than adequate power to provide authoritative results, the WASH arms of these trials were sadly without benefit in relation to the primary objective of reducing growth faltering. In this collection, Cumming et al. [11] summarise the results from these trials together with interpretative inferences from the senior investigators and a consensus statement. This statement makes a clarion call for so-called ‘Transformative WASH’ that provides comprehensive WASH inputs tailored to address the local exposure landscape and enteric disease exposure, and this call is supported by WaterAid [12].

The collection evaluates the longer-term consequences of childhood stunting, which include impaired cognitive and behavioural development. This relationship is explored by Xie et al. [13] using neuroimaging tools and sensitive behavioural assays to explain exactly how stunting impacts brain development. Their study involves two longitudinal cohorts of infants and toddlers recruited from an urban slum in Dhaka, Bangladesh, and shows, for the first time, that brain functional connectivity may serve as a neural pathway by which biological adversity impacts cognitive development. These findings advance our understanding of the neural pathways that may be involved in linking the relationship between growth faltering and poor cognitive outcome. This new knowledge should advance the development of more efficient interventions.

As noted in the introduction, the prevalence of wasting and stunting has declined to some extent over the past few decades; however, prevalence of obesity has steadily increased. In this collection, the epidemiological burden of childhood obesity is clearly highlighted [14]. Data show that for children aged 5 to 19 years, global obesity was relatively rare in 1975, at 0.7% in girls and 0.9% in boys, but by 2016 it had reached 5.6% in girls and 7.8% in boys. This study identifies the key drivers of this epidemic as the changing food systems and reduced physical activity, and clearly calls for the implementation of significant programmes and policies in multiple sectors to address over-nutrition, undernutrition, mobility and physical activity. The results regarding the impact of changing food systems are well aligned with a study by Jia et al. [15], which found that the school neighbourhood food environment in the USA—such as an increase in the number of convenience stores and fast-food restaurants close to schools—may affect obesity risk in young children.

The role that childhood stunting plays in the development of overweight and obesity in later life is still not well understood. This has mainly been because few studies follow up children to adulthood and the current anthropometric measures of nutritional status may not be sufficiently robust for all purposes [16]. Estimating body composition of malnourished children at baseline, during treatment and long after treatment may provide additional understanding into this relationship. In this collection, we share a narrative review on body composition methods that can be used for undernourished children and a summary of published data on this topic [16]. From these data we learn that all forms of undernutrition adversely impact fat-free mass, reduce adiposity and contribute in the long term to elevated non-communicable disease risk.

## Conclusion

Nutrition scientists and policymakers trying to understand and hence address the multiple burdens of malnutrition in all its causes are bedevilled by frustrations and yet blessed with some successes. The successes generally occur without the aid of targeted interventions; by this, we refer to the fact that many forms of undernutrition (stunting, wasting, anaemia, vitamin A deficiency) resolve as nations pass through the demographic and wealth transition [16]. This is the good news. The bad news is that in almost all such circumstances the pendulum swings too far and the population races towards an obesity epidemic: Mexico has been a prime example [17]. The other bad news is that specific interventions to address stunting in countries that are not blessed by rapid economic progress are often stubbornly unsuccessful, for example, see the summaries of interventions against EED and the large WASH interventions summarised here. The search for integrated and affordable solutions must go on.

We hope you enjoy this collection as much as we enjoyed helping to compile it.

## Abbreviations

EED: Environmental enteric dysfunction
LMIC: Lower- and middle-income counties
WASH: Water, sanitation and hygiene
WHO: World Health Organization
